# A Novel Mutation of the KLK6 Gene in a Family With Knee Osteoarthritis

**DOI:** 10.3389/fgene.2021.784176

**Published:** 2021-11-11

**Authors:** Yanzhi Ge, Chenfen Zhou, Xiujuan Xiao, Zhijiang Jin, Li Zhou, Zuxiang Chen, Fucun Liu, Qiang Yuan, Guoqing Zhang, Letian Shan, Peijian Tong

**Affiliations:** ^1^ The First Affiliated Hospital, Zhejiang Chinese Medical University, Hangzhou, China; ^2^ National Genomics Data Center, CAS Key Laboratory of Computational Biology, Bio-Med Big Data Center, Shanghai Institute of Nutrition and Health, University of Chinese Academy of Sciences, Chinese Academy of Sciences, Shanghai, China; ^3^ College of Pharmaceutical Sciences, Zhejiang Chinese Medical University, Hangzhou, China; ^4^ Department of Orthopaedics, The 9th People′s Hospital of Hangzhou, Hangzhou, China; ^5^ Department of Orthopaedics, Changzheng Hospital, Second Military Medical University, Shanghai, China

**Keywords:** KLK6, IL6, knee osteoarthritis, mutation, whole-exome sequencing

## Abstract

To investigate the correlation between gene mutation and knee osteoarthritis (KOA), a whole-exome sequencing (WES) was applied to analyze blood samples of four KOA patients and two normal subjects in a family. Gene mutations were identified by gene-trapping and high-throughput sequencing analysis across the differences between the patients and normal subjects. The interactive gene network analysis on the retrieval of interacting genes (STRING) database and the KOA-related genes expression data sets was performed. A possibly detrimental and nonsynonymous mutation at the kallikrein-related peptidase 6 (KLK6) gene (rs201586262, c. C80A, P27H) was identified and attracted our attention. KLK6 belongs to the kallikrein family of serine proteases and its serum level is known as a prevalent biomarker in inflammatory and malignant diseases. KLK6 expresses in the extracellular compartment for matrix degradation, highlighting that KLK6 plays a role in the pathogenesis of KOA. By using the gene databases, the KOA-related genes were mined after de-duplication and IL6 was selected as the most relevant gene through interactive analysis of protein-protein interaction (PPI) network. The data suggested that KLK6 gene mutation and the related expression alteration of IL6 gene might determine the occurrence of hereditary KOA. The is the first study discovering the gene mutation of KLK6 as a factor of pathogenesis of KOA, especially the hereditary KOA.

## Introduction

Knee osteoarthritis (KOA) is the most common and disabling joint disease, affecting up to 25% of people over the age of 50 years worldwide (Goff and Elkins, 2021). It is associated with degeneration of whole joint components, such as cartilage, subchondral bone, synovial membrane, periarticular muscles, and para-articular tendons, resulting in joint swelling, pain, and dysfunction (Slomski, 2020). Given the high disease burden and impact on health care systems, there is a need to develop effective remedies for KOA (van Tunen et al., 2018; Moghimi et al., 2019). Currently, the strategies used for KOA management mainly focus on relieving pain, decreasing inflammation and enhancing functional improvement through physiotherapy, pharmacotherapy, and end-stage surgery (Arden et al., 2021). The etiology and pathogenesis of KOA are driven by a variety of factors. Environmental stress and genetic mutations are two main pathogenic factors of KOA (Magnusson et al., 2019). For instance, obesity-induced biomechanical overload on joints results in periarticular trauma and joint deformity constituting the mechanical pathogenesis of KOA. Recent studies (Wilkinson, 2021) have shown that genetic mutations in cartilage matrix proteins can induce the onset of KOA lesions. This suggests that KOA may be a hereditary disease in some cases. However, this has not been clarified to date.

Genetic factors, such as heritable and somatic mutations, contribute to the development and progression of many diseases. Thus, understanding the genetic heterogeneity of diseases is a necessary prerequisite for accurate diagnosis and precise application of therapy. Gene sequencing technologies have been developed to identify pathogenic genes, hence provide new perspectives into the molecular mechanisms involved in hereditary diseases (Wang et al., 2018). For instance, the whole-exome sequencing (WES) method is routinely used as a diagnostic tool. Generally speaking, WES is a genetic testing method that describes an individual’s entire exon makeup (Karapetis et al., 2008; Silva et al., 2017; Wise et al., 2019). This makes WES an ideal method for analyzing the potential genetic mechanisms of malignancies, such as familial breast cancer and pancreatic cancer, based on high-throughput genomic data (Petersen et al., 2017). Previous research has revealed one shared variant (rs3732378) at position 280 in the transmembrane domain of CX3CR1 in four severely affected family members of developmental dysplasia of the hip with the help of WES, with the result that caused a threonine (polar) to methionine (non-polar) alteration (Feldman et al., 2013). For non-neoplastic diseases, this method can be used to confirm the diagnosis of genetic disease as well as to assess genetic risk and predict drug responses. A previous study (Min et al., 2007) identified 9 novel variants contributing to the early-onset of KOA, 2 (IDH1 Y183C and NRP2) of which were promising. These two variants (NRP2 c.1938–21 T > C and IDH1 c.933–28C > T) occurred together on haplotypes with radiographic signs of KOA in two out seven families. Further mutation analysis of the linkage area on chromosome 2q33.3-2q34 may reveal variants involved in advanced KOA. Thus, WES presents a promising method for the diagnosis of hereditary diseases.

Here, by using WES, the KOA-related mutations were detected from six members (four KOA patients and two healthy subjects) of a family. The STRING database and the gene databases (GeneCard database, Pharmacogenomics Knowledgebase (PharmGKB) database, Online Mendelian Inheritance in Man^®^ (OMIM) database, and DrugBank database) were used for discovering potential connections between the mutations and KOA. The mutation of KLK6 (rs201586262, c. C80A, P27H) was identified as the most relevant gene mutation to the KOA family. This is the first study on the relationship between KLK6 mutation and KOA.

## Materials and Methods

### Ethics Statement

All blood samples used in this study were acquired by the Ethical Committee of the First Affiliated Hospital, Zhejiang Chinese Medical University. Besides, the study was conducted in accordance with the Declaration of Helsinki and informed consent was acquired from all participants.

### Sample Collection

Blood samples of four KOA patients and two normal subjects were collected from one family in the First Affiliated Hospital, Zhejiang Chinese Medical University. All patients were Chinese and had been diagnosed by the American College of Rheumatology (ACR) Diagnostic Criteria for Knee Osteoarthritis (1995) and the Standards for Diagnosis and Treatment of Traditional Chinese Medicine (1994). Patients who were treated with other drugs and other methods within a week, caught tumor-related diseases and severe inflammatory diseases, or underwent surgery at joints were excluded. We collected 4 ml of blood for each person and all samples were stored at −80°C with dry ice transportation.

### DNA Isolation and Storage

Approximately 3 ml blood samples from all six participants were collected in EDTA-coated centrifugal tubes and DNA was extracted as previously described (Bulla et al., 2016). Nanodrop 2000 (Thermo Scientific) was used for DNA quantification. For further analysis, extracted DNA from each specimen was first collected and then stored at −80°C.

### WES

WES was performed by the Illumina HiSeq3000 paired-end sequencing (Guangzhou Ruibo Biotechnology Co., Ltd.) after using Agilent SureSelect All Exon Targeting Kit V6+ Cosmic for libraries preparation.

### Variant Calling

The Genome Analysis Toolkit (GATK Version: 4.1.0.0, https://gatk.broadinstitute.org/hc/en-us) was used for variant discovery (Min et al., 2007). Raw reads were mapped to the hg38 human reference genome using Burrows-Wheeler Aligner (Version: 0.7.1) (Li and Durbin, 2010). Germline mutation was called using HaplotypeCaller in its default single-sample mode according to GATK. CNN score variants and filter variant tranches tools were both applied to filter variants for further analysis.

### Variation Annotations and Analysis

Functional annotations of variants were performed with ANNOVAR (Version 2019-10-24) using target databases of the hg38 human reference genome (Yang and Wang, 2015). After annotations, one coding variant was identified: 1) shared at least in three affected individuals; 2) absent in two unaffected individuals; 3) rare, with a minor allele frequency (MAF) of ≤1% in the 1,000 Genomes Project (1 KG), Exome Aggregation Consortium (ExAC) and Genome Aggregation Database (GnomAD); 4) nonsynonymous mutation; 5) possible detrimental mutation according to the default scores from SIFT (Sim et al., 2012), polyphen2 (Emadi et al., 2020) and FATHMM (Shihab et al., 2013).

### STRING Analysis

Protein-protein interaction (PPI) referred to the connecting process in which two or more protein molecules form protein complexes through noncovalent bonds using the STRING database (https://string-db.org/) (Athanasios et al., 2017). Briefly, the column of “protein by name” and the organism of “*Homo sapiens*” were chosen to construct a framework of a net. Then, the network type (full network), the meaning of network edges (evidence) and active interaction sources (including text mining, experiments, databases, co-expression, neighborhood, gene fusion, co-occurrence) were used as primarily basic settings. A confidence level of a minimum required interaction score of medium confidence (Value = 0.4) and a limitation of the max number of 20 interactors in the network were set to discover the potential connections. Finally, a protein interaction network was constructed, and then the graphic of the visual result was downloaded for further analysis.

### Targeted and Overlapped Genes Related to KOA

Using “Knee Osteoarthritis” as a keyword, the KOA-related genes were searched in the following four databases: GeneCard database (Stelzer et al., 2016) (https://www.genecards.org/), PharmGKB database (Whirl-Carrillo et al., 2012) (https://www.pharmgkb.org/), OMIM database (Amberger et al., 2015) (https://www.omim.org/), and DrugBank database (Wishart et al., 2018) (https://go.drugbank.com/). For the DrugBank database, the relevant score greater than or equal to 20 was enrolled in this study, whereas a value of greater than 80 will be deleted. For the GeneCards database, the relevant score greater than or equal to 10 was regarded as an inclusion criterion. Then, the KLK6-connected genes from the STRING database and overlapped KOA-related genes from the four databases were selected, and a visual diagram was constructed using an R package (Bayani and Diamandis, 2011).

## Results

### Participants Information

**FIGURE 1:**
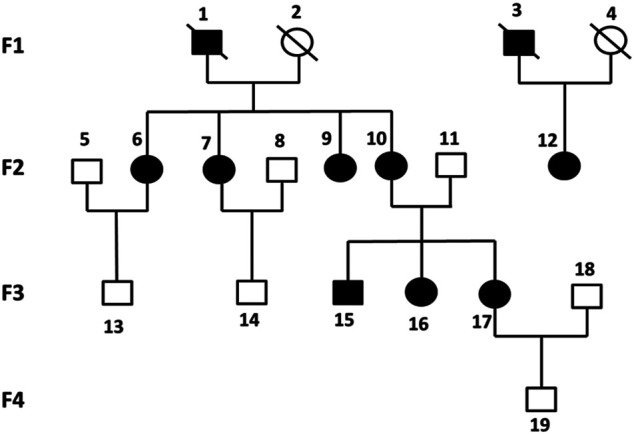
Pedigrees of all participants in four generations of this study. Each individual was represented by a circle (if female) or a square (if male). Black shaded symbols denoted subjects diagnosed with KOA. Abbreviation: KOA, knee osteoarthritis.

Six previously unreported Chinese subjects in a family were included in this study. Four subjects (F2-6, F2-7, F2-12, F3-15) were diagnosed with KOA, and the other two non-KOA subjects (F3-13, F3-14) acting as a control. The percentage of females was 75% in all patients and only male members were in the normal controls. The relationship between family members was depicted in Figure 1.

### Sequencing Analysis

**FIGURE 2:**
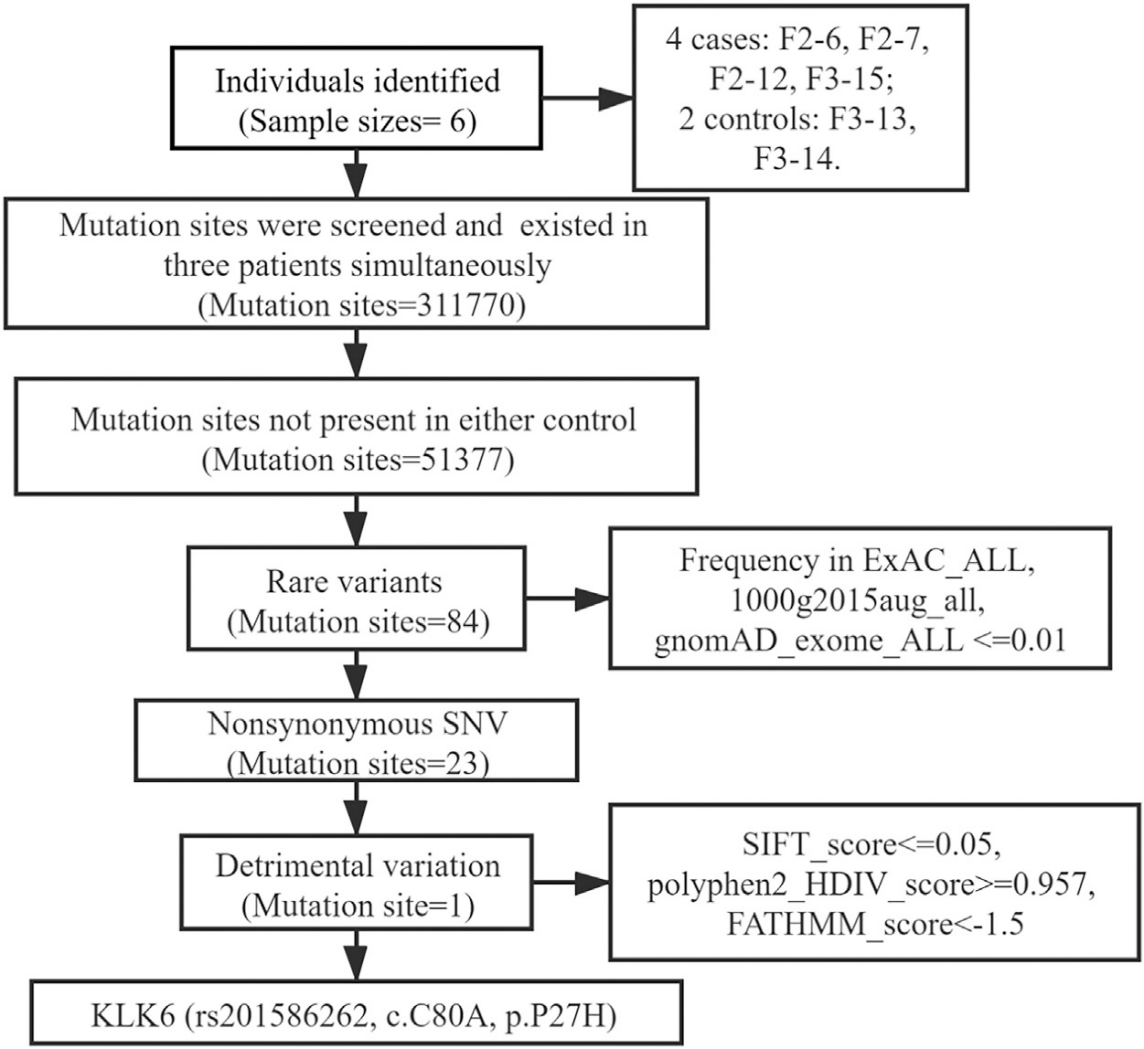
Flow chart of mutation sites selection.

**FIGURE 3:**
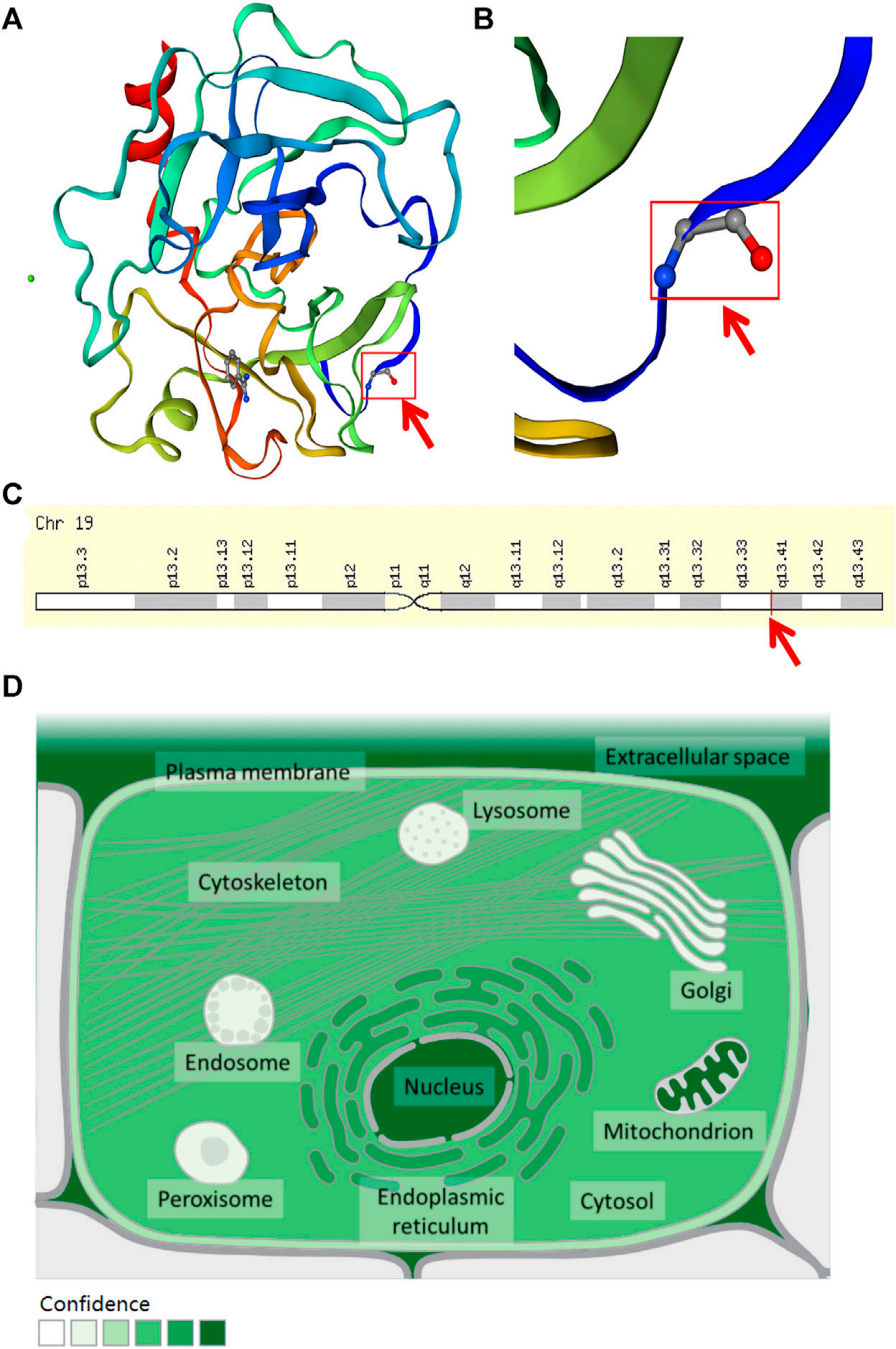
The location and distribution of KLK6. **(A)** The rs201586262 mutation of KLK6 protein. **(B)** The magnification of the rs201586262 mutational site. **(C)** The location of KLK6 gene on a chromosome. Note: Red color arrows represent a specific mutation site. **(D)** The distribution of KLK6 in the microenvironment. Note: The deeper the color, the higher confidence of the KLK6 expression.

The WES results revealed a total of 311,770 variants co-existing in at least three-quarters of KOA members. After deleting variants appearing concurrently in control specimens, 51377 variants were retained from all DNA sequence variations. Using the databases (ExAC and gnomAD) and 1000g2015aug for ANNOVAR, we set the minor allele frequency (MAF) less than or equal to 0.01 to acquire a rare mutation. After that, 84 variants were acquired for further analysis. Of these remaining variants, 23 variants were nonsynonymous, regardless of beneficial, harmful, or fatal to the expression products of genes. Finally, to further explore the key mutation and exclude beneficial mutation genes, we went on to screen the detrimental mutation in ANNOVAR results using the default parameters and ultimately the KLK6 gene was identified. The screening process of variants was depicted in Figure 2. As a result, we identified a potential nonsynonymous and detrimental mutation in the KLK6 gene (rs201586262, c. C80A, P27H), which occurred simultaneously in the same generation of F2-6, F2-7, and F2-12. However, the patient F3-15 in the next generation was not detected the same mutation. The accurate mutation site has been marked in the three-dimensional Figures 3A,B.

Besides, the KLK6 gene is located in the chromosomal region 19q13.3–13.4 (Figure 3C) and encoded for an enzyme with trypsin-like properties that can degrade the extracellular matrix (ECM) (Bayani and Diamandis, 2011). The result was consistent with the high expression in extracellular space (https://www.genecards.org/cgi-bin/carddisp.pl?gene=KLK6&keywords=KLK6) and shown in Figure 3D. Furthermore, the cytosine located at 80 in the KLK6 gene has mutated to adenine, leading to its encoded protein mutation from proline to histidine.

### PPI Network Construction and Databases Preparation

**FIGURE 4:**
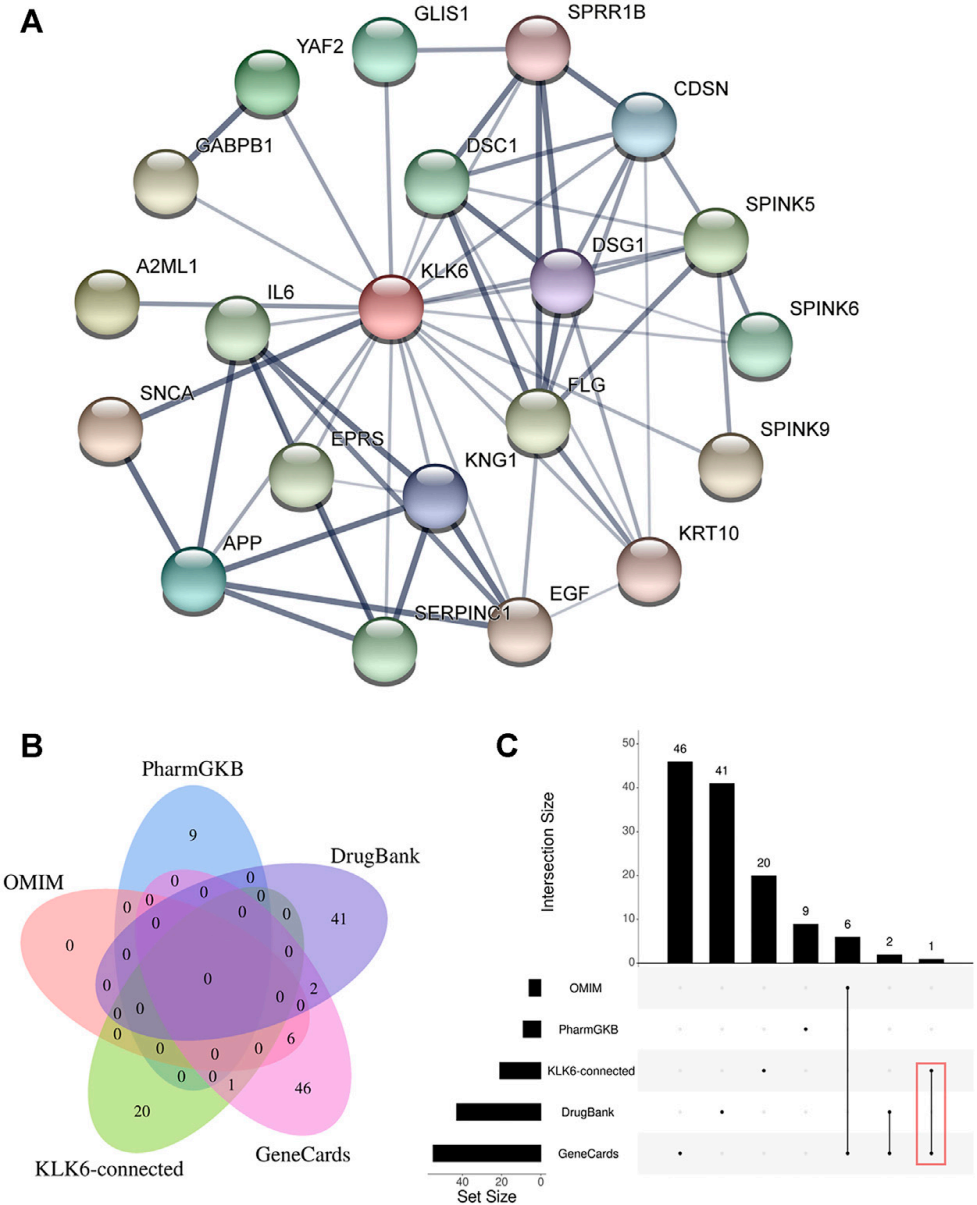
**(A)** PPI network of KLK6-connected genes. **(B)** The relationship between KLK6-connected genes and four KOA-related genes databases. **(C)** The demo diagram showed the relationship between KLK6-connected genes and other databases. Note: Red color marked the crossed genes. Abbreviation: PPI, protein-protein interaction; KLK6, kallikrein-related peptidase six; KOA, knee osteoarthritis; PharmGKB, Pharmacogenomics Knowledgebase; OMIM, Online Mendelian Inheritance in Man.

To further detect the KLK6-related gene, a PPI network was performed using STRING. As shown in Figure 4A, the result indicated that 20 most closely genes were related to KLK6, such as KNG1, EGF, SPINK9, SERPINC1, IL6, SNCA, YAF2, DSC1, KRT10, SPINK6, SPRR1B, FLG, SPINK5, DSG1, A2ML1, APP, EPRS, CDSN, GLIS1, and GABPB1.

Afterward, to further unravel the crossed genes, four databases were used to search KOA-related genes. After removing duplicates, a total of 105 KOA-related genes were identified by searching the GeneCard, OMIM, PharmGKB, and DrugBank databases, and the total gene names were provided in Table 1. After the overlapping analysis, we ultimately found a crossed gene (IL6) between KLK6-connected genes derived from the STRING and GeneCard database. The Venn diagram (Figure 4B) and a demo (Figure 4C) clearly pictured the relationship between KLK6-connected genes in the STRING database and multiple KOA-related genes in the other four databases.

**TABLE 1:** KOA-related genes in the database.

Databases	Count	Gene names
STRING database	20	KNG1, EGF, SPINK9, SERPINC1, IL6, SNCA, YAF2, DSC1, KRT10, SPINK6, SPRR1B, FLG, SPINK5, DSG1, A2ML1, APP, EPRS, CDSN, GLIS1, GABPB1
DrugBank database	57	NR1H3, NR1H2, ALOX5, CYP1A2, CYP3A4, CYP3A43, CYP3A5, CYP3A7, CYP2E1, CYP2C9, CYP2D6, PTGS1, PTGS2, TTR, CYP2C9, CYP2C19, CYP1A2, CYP2C8, ALB, ABCC4, ABCC1, SLC22A6, SLC22A8, SLCO1C1, SLC22A11, UGT2B7, CYP3A4, CYP2B6, CYP2C18, CYP2E1, SLCO1B1, ABCB11, UGT1A3, UGT1A9, UGT2B4, ALOX5, SCN4A, ASIC1, KCNQ2, KCNQ3, PLA2G2A, ABCB1, SLC6A4, SLC6A3, SLC6A2, CYP1A2, CYP2D6, CYP2C9, ALB, ORM1, ORM2, ABCB1, TRPV1, CYP1A2, CYP2C19, CYP2C9, CYP2E1
GeneCards database	55	COL2A1, COMP, ACAN, MATN3, COL9A1, SMAD3, COL9A2, GDF5, MMP13, SLC26A2, COL9A3, COL11A2, TGFB1, FBN1, RUNX2, FRZB, TRPV4, DDR2, COL10A1, COL11A1, IL1B, TNF, IL6, TGFB3, IL10, PTGS2, UFSP2, MMP3, CANT1, TRAPPC2, ASPN, FGFR3, CCN6, TGFB2, MMP1, COL5A2, TGFBR1, GALNS, IL1RN, COL1A1, TGFBR2, CRP, CXCL8, IL17A, TNFSF11, ALB, CHST3, MMP2, TNFRSF11B, BGLAP, MMP9, IL1A, TIMP1, SMAD4, COL5A1
OMIM database	3	ACAN, ASPN, COL2A1
PharmGKB database	9	CCAL1, OAP, OMD, TINAGL1, SLEN1, SLEN2, SLEN3, TINAG, GPNMB

Abbreviation: KOA, knee osteoarthritis; STRING, the search tool for the retrieval of interacting genes; OMIM: Online Mendelian Inheritance in Man; PharmGKB, pharmacogenomics knowledgebase

## Discussion

KOA is a chronic fading joint disease and is characterized by degeneration of articular cartilage in multiple locations (Iagnocco and Naredo, 2012). The pathogenesis of KOA has been linked to multiple mechanisms, such as agedness, obesity, gender, heredity, sports injury, inflammation, and some metabolic factors (Sandell, 2012). In recent years, studies have uncovered new molecular pathogenic factors of KOA (Chu et al., 2017; Casalone et al., 2018). Previous studies have found a positive association of two AKNA polymorphisms (rs10817595 and rs3748176) with KOA from a blood DNA bank of 181 KOA patients and 140 healthy controls (Martínez-Nava et al., 2018). Analysis of genetic variations in 3217 KOA patients and 2,214 healthy controls revealed a nonsynonymous ADAMTS14 polymorphism (rs4747096) significantly associated with KOA in females (Rodriguez-Lopez et al., 2009). Meanwhile, liquid biopsy showed great potential in disease detection and the research on blood gene expression in KOA become a hot topic. Through a systematic review of the literature, Luo et al. (Luo et al., 2019) found that the deficiency of G protein-coupled receptors induced osteoporosis, osteoarthritis, and delayed fracture healing. In this study, blood samples were obtained from patients with familial KOA and two normal members who served as the control group. To our knowledge, none has reported the role of gene mutations in familial KOA. Herein, we investigated the role of gene mutations in KOA inheritance. Based on the WES, a detrimental mutation (rs201586262, c. C80A, p. P27H) in the KLK6 gene was found in three patients (F2-6, F2-7, F2-12) of the same generation. A total of 124 SNPs (100 genes) and 105 SNPs (104 genes) were respectively reported to be significantly associated with KOA risk (Aubourg et al., 2021; Boer et al., 2021). However, none of the reported SNPs were found in our data. About 173 genes were found both present in our study and other publications (Supplementary Table 1). By filtering the minimum allele frequency (≤1%), these SNPs and genes were all eliminated.

The human tissue kallikreins (KLKs) were an important family of about 15 serine proteases that regulate the proteolysis of endogenous substrates (Yousef and Diamandis, 2001). KLKs genes were localized on chromosome 19q13.4 and have been implicated in various physiological and pathological processes (Borgoño and Diamandis, 2004). KLK6 was a 223-amino acid residues serine protease expressed in multiple tissues and organs (Petraki et al., 2001). The results of PCR showed that KLK6 is expressed in the prostate, kidney, endometrium, brain, and spinal cord (Anisowicz et al., 1996; Yamashiro et al., 1997). Using the ELISA test, antigens against KLK6 were found in breast cyst fluid, male and female serum and milk (Diamandis et al., 2000). The immunohistochemical assay revealed that KLK6 was localized in normal human tissues (Petraki et al., 2001). It has been reported that the KLK6 gene could be cloned independently of other genes, such as zyme in brain tissue (Little et al., 1997), protease M in breast tissue (Anisowicz et al., 1996), and neurosin in a colon carcinoma cell line (Mitsui et al., 2002). Other studies demonstrated that KLK6 played an important role in many non-neoplastic diseases, such as inflammatory and degenerative illnesses, as well as trauma lesions of the central nervous system (Silva et al., 2017). Evidence from prior studies demonstrated that the KLK6 gene and its expressed products might regulate the degradation of β-amyloid or turnover of amyloid precursor protein (Ogawa et al., 2000). Most recent works (Yousef et al., 2003) have shown that the expression of this gene was significantly elevated in cancers, such as ovarian cancer, but studies on the expression of this gene in arthritis were few. Previous studies have also shown that blood KLK6 concentration was influenced by the advanced age and underlying neurologic pathology. Ghosh et al. (Ghosh et al., 2004) found that hK6 (also known as KLK6) was involved in matrix protein degradation and degradation of high-molecular-weight ECM proteins such as fibronectin, laminin, vitronectin and collagen.

The ECM was recognized as an active entity composed of hydrated macromolecular proteins and sugars (Akhmanova et al., 2015). Actually, ECM contained adhesive proteins, notch signaling molecules, and proteoglycans, all of which regulated and modulated various activities (Sánchez-Romero et al., 2019). The ECM connected with the body matrix and formed a major component of tissues. Basically, cells were surrounded by ECM which was an organized spatial network providing both structural and biochemical support to the cells. In this study, the results showed that cytosine located on the number 80 of the KLK6 gene was mutated to adenine, resulting in a change from proline to histidine and denaturation of the KLK6 protein. This mutation-induced change may increase the binding ability of KLK6 to its substrates during catalytic process, and as a result, this promoted the degradation of the extracellular matrix. Chondrocytes that secreted and formed the main components of ECM played an important role in maintaining joint homeostasis. In KOA, the equilibrium between synthesis and degradation of ECM was disrupted leading to remodeling of corresponding articular cartilage tissues (Rahmati et al., 2017). This indicated that the KLK6 gene may indirectly negatively influence KOA.

The STRING was adopted to construct PPI networks. The STRING database, complemented with computational predictions, was aimed to collect, score and build PPI networks (Szklarczyk et al., 2019). The PPI contributes to a better understanding of the interactive internet of target genes or proteins. Hence, it was significant to integrate all PPIs under one framework, and visualization of networks was necessary to provide data analysis using pipelines in diverse areas (Khurana et al., 2017; Huang et al., 2018). In this study, a total of 20 genes that were closely related to KLK6 were identified in the PPI. The proteins encoded by these genes were predicted to play a role in the development of KOA. To further explore the roles of these genes in KOA, we analyzed the four most commonly used databases to determine whether there was an intersection or not. Interestingly, analysis of data from the GeneCards database revealed that IL6 and KNG1 were overlapping genes.

GeneCards was a searchable, integrative database that could provide us with comprehensive information and be beneficial to predict human disease genes. Meanwhile, this database could provide us with gene-centric data from multiple sources, such as genetic, transcriptomic, genomic, proteomic, clinical, and functional information (Stelzer et al., 2016). As a result, the screening data provided by GeneCards may indicate a crucial means for associating diseases with their causative genes. In this study, the IL-6 in the GeneCards database was found and the connection of the PPI network may reveal a significant meaning for KLK6. Besides, numerous studies have revealed a connection between IL-6 and KOA. For instance, IL-6 was significantly increased in the articular synovial membrane, subchondral bone, or cartilage of KOA patients, confirming its roles in KOA pathogenesis (Wang and He, 2018). Previous studies (Greene and Loeser, 2015) had revealed that increasing IL-6 levels in the blood could significantly reduce the patients’ physical function. Meanwhile, elevated IL-6 had been linked to increased risk of KOA progression. A recent clinical study covering 33 patients with different Kellgren-Lawrence grades showed that IL-6 and IL-10 were significantly higher in both serum and synovial fluid of KOA patients compared with samples from normal control (Sachdeva et al., 2019).

This article had some limitations: (a) if the information and samples of all members in the enrolled family with KOA were available at the beginning of the study, then a WES could be performed for all family members. (b) the lack of complete patient medication records made it impossible to determine whether the missense mutation of KLK6 interfered with drug efficacy. (c) given the lack of direct trios (father, mother, child) relationship between the members, the transmission disequilibrium test could not be used to conduct family-based analysis. (d) the risk of bias was inevitable because of the small number of samples involved.

In spite of these limitations, our results added values to the understanding of the pathogenesis, accurate diagnosis and targeted therapy, as well as classification of a new hereditary-related KOA subtype. In the future, we will conduct animal and molecular experiments to verify the role of KLK6 in KOA.

## Conclusion

WES analysis of blood samples identified a detrimental mutation of KLK6 (rs201586262, c. C80A, P27H) gene that may contribute to the development of KOA in a family. Analysis of four gene-related databases revealed that IL6 gene was overlapped with KLK6 in KOA. This study demonstrated for the first time that mutation of the KLK6 gene might modulate the development of KOA, especially the hereditary KOA, which still need futher verification by experiments.

## Data Availability

All data can be viewed in NODE (http://www.biosino.org/node) by the accession number OEP000641 or through the URL: http://www.biosino.org/node/project/detail/OEP000641.
